# Short-term outcomes of robotic vs. laparoscopic surgery for gastric cancer after neoadjuvant therapy: a systematic review and meta-analysis

**DOI:** 10.1186/s12885-025-14395-3

**Published:** 2025-06-05

**Authors:** Tuerjun Tuohuti, Kamuran Abulizi, Tao Li

**Affiliations:** 1 Medical Treatment Center of Gastrointestinal Surgery, People’s Hospital of Xinjiang Uygur Autonomous Region, No. 91 Tianchi Road, Tianshan District, Xinjiang, 830011 Urumqi China; 2 Xinjiang Medical University, No. 91 Tianchi Road, Tianshan District, Xinjiang, 830011 Urumqi China

**Keywords:** Robotic gastrectomy, Laparoscopic gastrectomy, Gastric cancer, Neoadjuvant therapy, Meta-analysis

## Abstract

**Background:**

The impact of robotic gastrectomy (RG) surgery on advanced gastric cancer following neoadjuvant therapy remains a topic of debate. A thorough search and analysis of the current relevant evidence is needed. This study aims to evaluate the efficacy, safety, and advantages of RG for gastric cancer after neoadjuvant therapy, comparing it with traditional laparoscopic gastrectomy (LG) surgery.

**Methods:**

We searched databases,including PubMed, Embase, Web of Science,Cochrane Library, and Chinese National Knowledge Infrastructure(CNKI),to identify studies up to May 10, 2025. Four non-randomized controlled trials from East Asia involving neoadjuvant therapy for advanced gastric cancer with RG and LG interventions were included. The outcomes assessed include: postoperative complications, operative time, blood loss, postoperative hospital stays, number of lymph node dissections, the first flatus, the first time on liquid diets, re-admission within 30 days after surgery, reoperation within 30 days after surgery, open conversion, prevalence of serious complications.

**Results:**

A total of four studies enclosed by 569 participants were incorporated into the analysis. The findings reveal that RG significantly extended operative time [mean difference(MD): 82.16,95%CI: 65.39 to 98.94, *P* < 0.00001, I^2^ = 30%] when compared to LG.; However, it significantly reduced the time to the patient's first flatus (MD: -0.60,95%CI:-0.70 to-0.51, *P* < 0.00001, I^2^ = 0%)and the first time on liquid diets[MD:1.33,95%confidence interval(CI):-1.51to-1.16, *P* < 0.00001, I^2^ = 0%], while also increasing the number of lymph nodes(MD: 1.76;95%CI:0.26to3.26, *P* = 0.02, I^2^ = 0%). Furthermore, the findings of this study demonstrate that there were no statistically significant differences between the RG and LG,with postoperative complications [odds ratio, OR: 0.81;95%CI: 0.35–1.87, *P* = 0.62, I^2^ = 65%], blood loss(MD: 2.34;95%CI: -6.43to11.10, *P* = 0.60, I^2^ = 0%), open conversion(OR: 0.66;95%CI: 0.18–2.38, *P* = 0.52, I^2^ = 0%), postoperative hospital stays(MD: -0.29;95%CI:-0.72to0.15, *P* = 0.19, I^2^ = 29%), reoperation within 30 days after surgery(OR: 0.49;95% CI:0.09,2.73, *P* = 0.42, I^2^ = 0%), re-admission within 30 days after surgery(OR: 0.59; 95% CI: 0.18,1.93, *P* = 0.38, I^2^ = 0%), and prevalence of serious complications(OR = 0.61, 95% CI: (0.29, 1.24), *P* = 0.17, I^2^ = 0%).

**Conclusion:**

Based on available data suggests that robotic surgery after neoadjuvant therapy is a treatment approach with great potential for development and may be used as a new treatment method for locally advanced gastric cancer.

**Trial registration:**

https://www.crd.york.ac.uk/PROSPERO/view/CRD42025643235, PROSPERO (42,025,643,235).

**Supplementary Information:**

The online version contains supplementary material available at 10.1186/s12885-025-14395-3.

## Introduction

The rising rates of new cases and deaths from gastric cancer are becoming a significant global health concern. Gastric cancer is the second leading cause of cancer-related death worldwide, with increasing diagnoses and mortality rates [1, 2]. In the comprehensive treatment of gastric cancer, radical surgery is a crucial component of the therapeutic approach [3].Specifically, neoadjuvant combined surgical treatment has gradually become the first-line treatment for advanced gastric cancer [4, 5]. To improve the prognosis, perioperative chemotherapy or radiotherapy has been applied to patients with advanced gastric cancer, especially in Western countries [6–8].In 2006, Professor CUNNINGHAM D et all pioneered the adoption of neoadjuvant combined surgical treatment for patients with resectable gastric cancer. Improving the progression-free survival and overall survival rates for gastric cancer patients. [9] Several randomized controlled trials(RCTs) in East Asia have shown that 2–4 cycles of preoperative neoadjuvant therapy in locally progressive gastric cancer can reduce tumor stage, regress the tumor, and achieve complete pathologic response(pCR) [10, 11]. This can improve the R0 resection rate and 5-year survival [12–14]. But neoadjuvant therapy also has drawbacks, including tissue fibrosis, weakening of blood vessels, and loss of anatomical structures and planes, which raise a new challenge for surgery [15].The safety and feasibility of laparoscopic surgery after neoadjuvant therapy for advanced gastric cancer has been demonstrated in currently available studies [16–18]..However, laparoscopic surgery has drawbacks, including: lack of 3D visualization, stiff instruments, magnified field of view and trembling, long learning curve, and high demands on assistants. RG has developed rapidly in the field of gastrointestinal surgery in recent years and is expected to replace LG. RG has several advantages: it provides a stable, magnified 3D field of view, precision and flexibility in the operating plane [19]. There are a large number of studies about LG, but studies on the use of robotic surgery for gastric cancer after neoadjuvant therapy are currently very scarce. Whether to perform robotic surgery on advanced gastric cancer after neoadjuvant therapy remains a topic of controversy. To address the lack of high-quality evidence from current studies, we conducted a comprehensive literature search and meta-analysis of the most recent data published to date to assess the differences in short-term outcomes between RG and LG in gastric cancer after neoadjuvant therapy.

## Methods

This study was grounded in the Preferred Reporting Items for Systematic Reviews and Meta-Analyses (PRISMA) statement [20]. Institutional review approval and informed consent were not required, as we collected data directly from previously published studies.

### Search strategy

#### Computer search strategy

Computer search was conducted in multiple prominent databases, including PubMed, Embase, Web of Science and Cochrane Library,CNKI.The search was performed with a broad timeframe encompassing all available databases. The latest date to update search was May 10, 2025. We used the terms"stomach neoplasm"and"laparoscopic,""robotic,"and"neoadjuvant chemoradiotherapy"as keywords in our search strategy, employing a combination of full text and medical subject headings (Mesh). The detailed search strategies shown in (Supplementary Table 1). No linguistic restrictions were implemented. A thorough search was conducted of the references for the relevant reviews and included studies.

#### Manual search strategy

Searched the relevant authoritative journals and specialized books, such as the Chinese Journal of Gastrointestinal Surgery, Chinese Journal of Robotic Surgery, Chinese Journal of Minimally Invasive Surgery, Chinese Journal of Laparoscopic Surgery, China Journal of General Surgery.

### Study selection

#### Eligibility criteria

The preselected PICO criteria: (1) patients: Patients who have undergone radical surgery for gastric cancer after neoadjuvant therapy (2) intervention: intervention with robotic gastrectomy (3) comparator: compare with laparoscopic gastrectomy (4) Outcome: Primary outcome: postoperative complications, secondary outcome: operative time, blood loss, postoperative hospital stays, number of lymph node dissection, the first flatus, the first time on liquid diets, re-admission within 30 days after surgery, reoperation within 30 days after surgery, open conversion, prevalence of serious complications.

(5) Study design: randomized controlled trials or nonrandomized controlled trials. Exclusions criteria: (1) Reviews, conference abstracts, case reports, letters to the editor, scientific reports. (2) Single-arm studies and non-human studies. (3) Important data incomplete and inaccessible. (4) Trials with unclear efficacy results.

### Data extraction

Extracted including primary and secondary endpoints data and recorded independently by two evaluators (Tuerjun and Kamuran). A third evaluator (TL) made the final decision on any discrepancies that may have occurred between the two evaluators. Data extracted from each study included study type, country, sample size, age, body mass index (BMI), sex, first author, year of publication, intervention type, control group, and outcome, preoperative neoadjuvant therapy cycle, neoadjuvant therapy regimens, and extent of surgery (gastrectomy). If it was not possible to extract relevant information from the literature, we attempted to contact the corresponding author of the study to obtain the information.

### Quality assessment

Limited number of relevant research articles, no randomized controlled trials retrieved. ROBINS-I (Risk of Bias in Non-Randomized Studies of Interventions), a method to assess the risk of bias in non-randomized studies [21, 22]. The response options for the signaling questions were: “Not applicable”, “Yes”, “May be”, “May not be”, “No”, and “No information”. The following seven types of bias were judged: bias due to confounding, selection bias, bias in classification interventions, bias due to deviations from the intended interventions, bias due to missing data, bias in outcome measurement, and bias in outcome reporting. Each domain's judgment affects the overall bias risk judgment across all domains.

### Statistical analysis

Meta-analysis was performed using Review Manager 5.4.1 software (The Cochrane Collaboration, 2020; London, UK). When the median with range or interquartile range was reported in the study, the MD and the SD were calculated according to the formula suggested by Abbas A et al. [23]and Luo et al. [24] Dichotomous data were expressed as odds ratio (OR) and continuous variables as mean differences (MD),95% confidence interval (CI) was calculated for each effect size. Tests of heterogeneity between studies were assessed using I^2^statistics [25, 26]. If *P* > 0.05 and I^2^ < 50%, studies were considered homogeneous and meta-analysis was performed using a fixed-effects model. If *P* < 0.05 and I^2^ > 50%, studies were considered heterogeneous, and sources of heterogeneity were analyzed. If the source of heterogeneity could not be explained by clinical or methodological heterogeneity, meta-analysis was performed using a random-effects model, and sources of heterogeneity were analyzed by subgroup analysis that included (tumor stage, extent of surgery, or institutional experience).

## Results

### Selected studies

A total of 139 articles were identified in the initial search. Duplicates were excluded by software for 23articles, and for the remaining 116 articles, 53 studies were excluded after reading the article titles and abstracts. The remaining 63 articles were subjected to a thorough examination of their content, resulting in the exclusion of one article due to the absence of essential data, (letters to the editor, reviews, conference papers, case reports, and technical reports) 58 articles. In the end, four studies were eligible and were included in the meta-analysis (Supplementary Fig. 1).

### Study characteristics and quality assessment

**Fig. 1:**
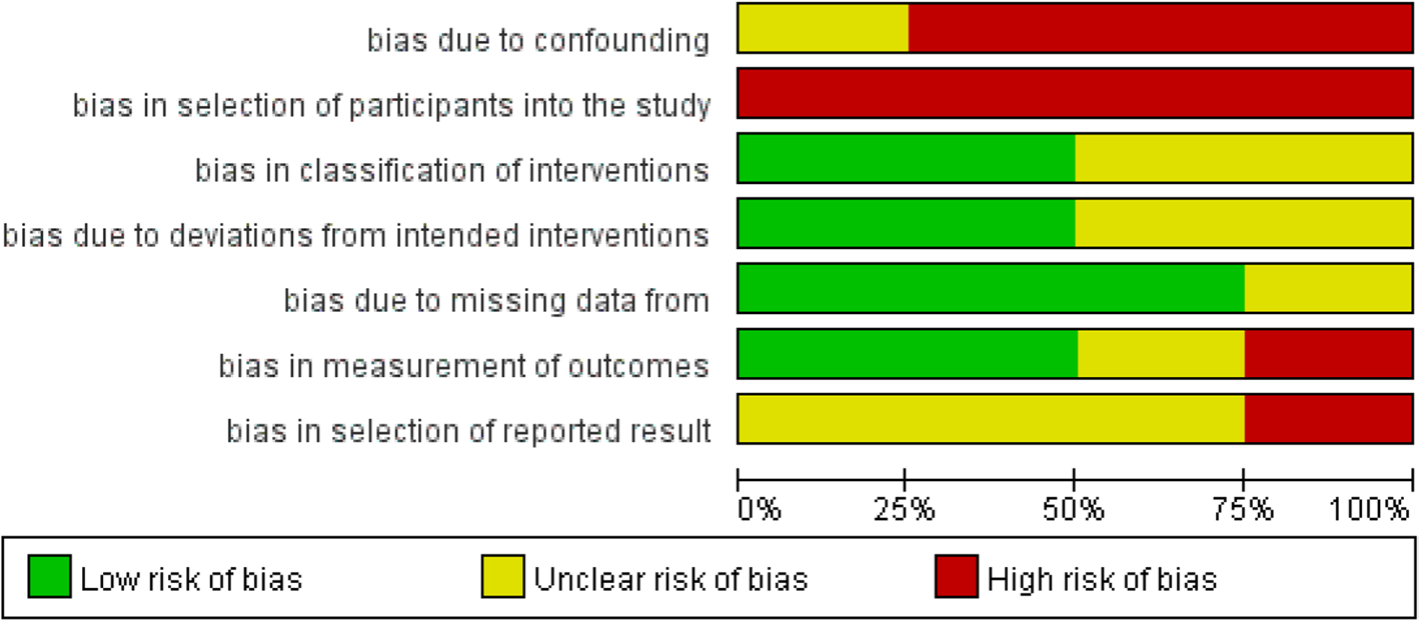
Risk of bias graph

**Fig. 2:**
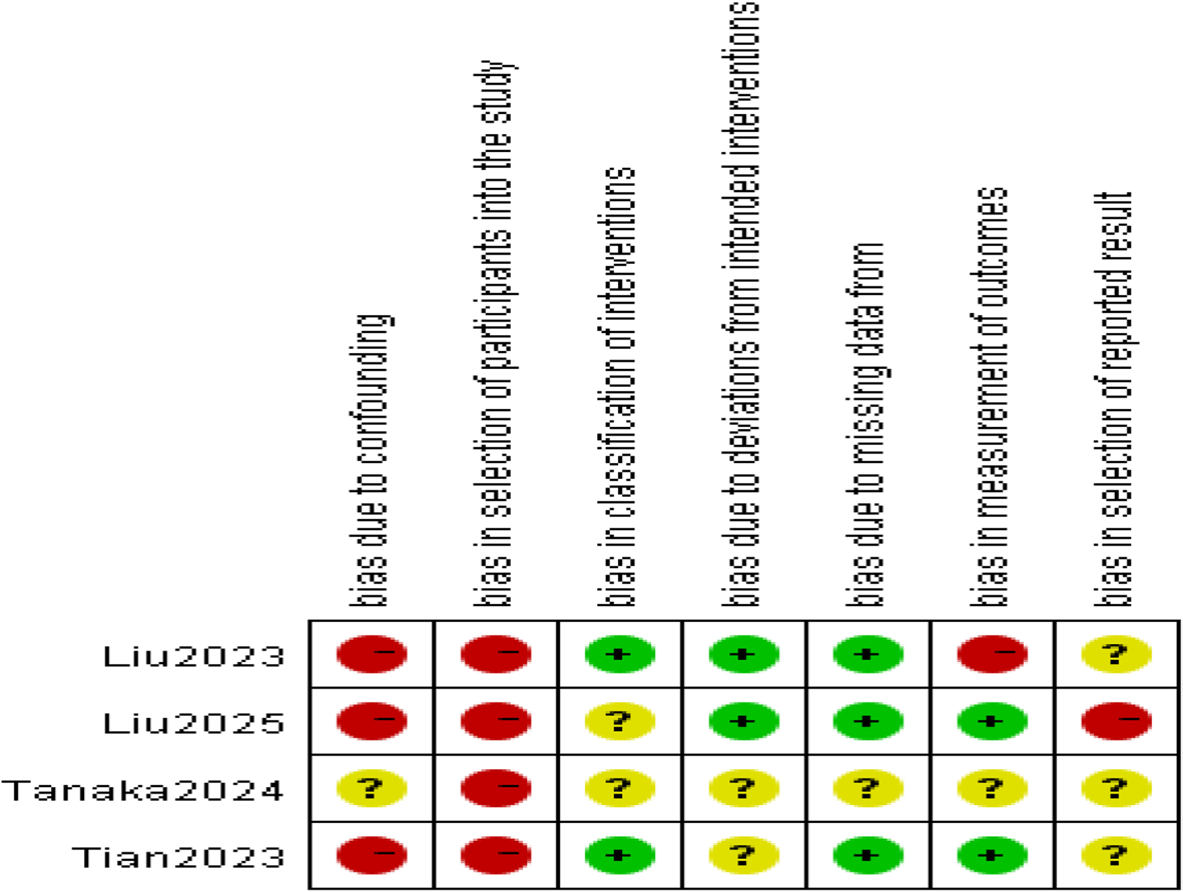
Risk of bias summary

All four included studies were non-randomized controlled trials, and two studies [27, 28] implemented propensity score matching. Four studies with a total of 569 participants (RG = 283, LG = 286), all trails occurred in East Asia from 2023 to2025.The study characteristics are shown in (Tables 1 and 2). The meta-analysis followed the Cochrane recommended methodology to assess the methodological quality of the included studies. The risk of bias judgments for the included studies are illustrated in the risk of bias graph (Fig. 1) and risk of bias summary (Fig. 2).

**Table 1:** Study characteristics

Author	Year	Study Design	Country	Age(years)	BMI (kg/m^2^) (R/L)	Preoperative neoadjuvant therapy cycle
Liu et all	2023	retrospective cohort study	China	R:66(58,71) L:65(57,69)	R: 22.76 ± 3.9 L: 21.93 ± 3.0	2
Liu et all	2025	PSM, retrospective cohort study	China	R:62.45 ± 10.1 L:61.71 ± 9.77	R: 24.34 ± 4.1 L: 23.97 ± 3.0	2–4
Tanaka et all	2024	retrospective cohort study	Japan	R: 66(59–71) L:66(59–71)	R: 21.8(19.8–24.1) L:21.8(19.8–24.1)	2–3
Tian et all	2023	PSM, retrospective cohort study	China	R:59.2(40–69) L:57.4(38–66)	R: 24.1 ± 4.6 L: 25.4 ± 5.9	2

*PSM* Propensity score matching; *BMI* body mass index

**Table 2:** Study characteristics

Sample (R/L)	Gastrectomy	Outcome			Neoadjuvant therapy regimens
60/60	DG/TG/PG	①②③④	⑤⑥⑨	⑦⑧	SOX
106/106	DG/TG/PG	①②③④⑤⑥ ⑪	⑦⑧⑨	⑩	SOX,XELOX,DOS,FLOT
50/53	DG/TG/PG	①②③④⑤ ⑪			SOX
67/67	DG	①②③④⑥⑦ ⑪	⑧⑩		SOX

SOX, S-1 plus Oxaliplatin; XELOX, Xeloda Plus Oxaplatin; DOS,Docetaxel Plus Oxaplatin Plus S-1;FLOT, Fluorouracil (5-FU) Plus Leucovorin Plus Oxaliplatin Plus Docetaxel. ①postoperative complications②operative time③blood loss④postoperative hospital stays⑤number of lymph node dissection⑥the first flatus⑦the first time on liquid diets⑧re-admission within 30 days after surgery⑨reoperation within 30 days after surgery⑩open conversion ⑪prevalence of serious complications

### Meta-analysis

#### Postoperative complications

**Fig. 3:**
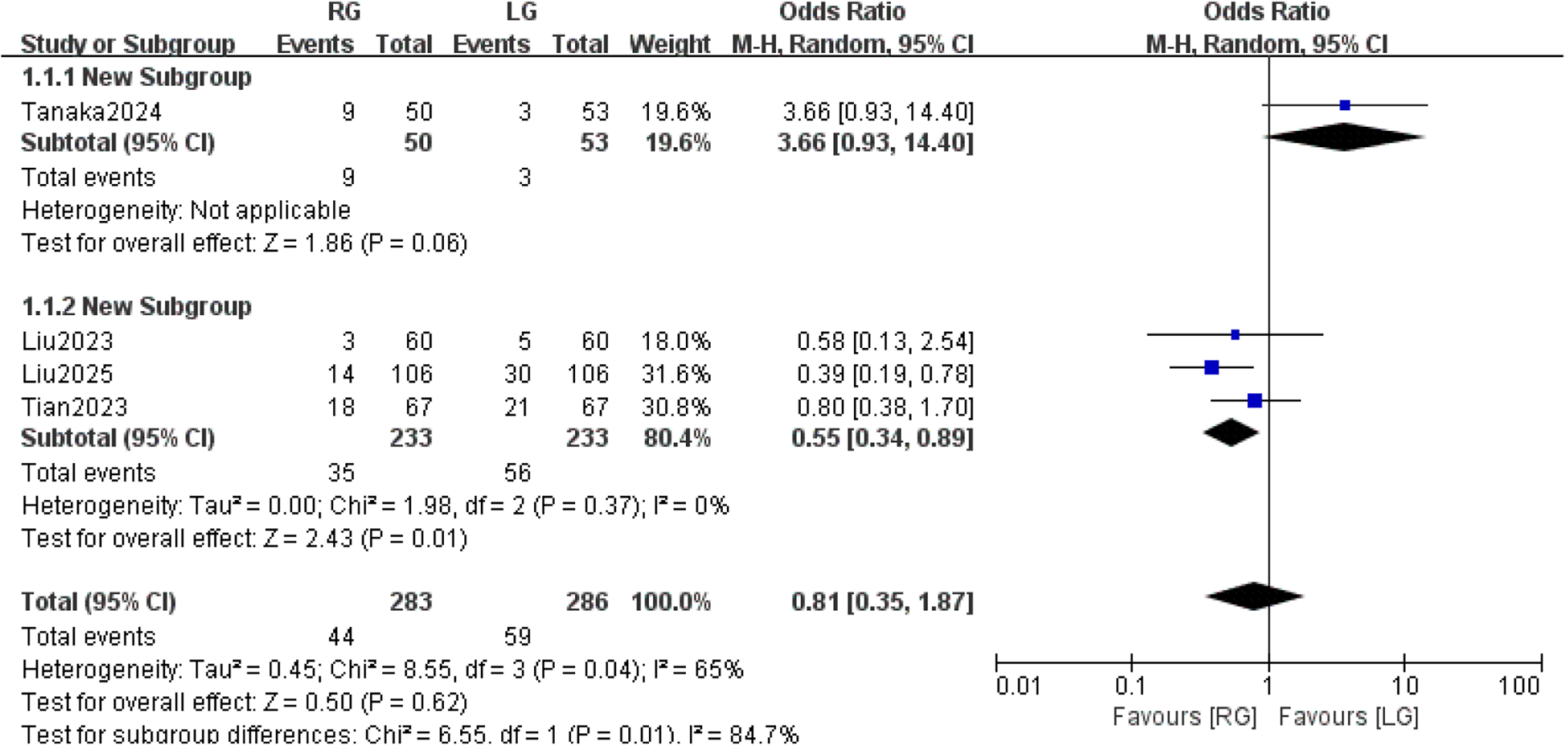
Postoperative complications

A total of 569 patients participated in four studies [27–30] mentioned postoperative complications. The results of the heterogeneity test showed statistically significant heterogeneity among the studies (*P* = 0.04, I^2^ = 65%), using a random effects model. In addition, it is already well known that institutional surgical experience is one of the main risk factors for the development of postoperative complications. The results of the subgroup analysis showed: [OR = 0.551, 95% CI: (0.34, 0.89), *P* = 0.01]. Institutional surgical experience may be a potential source of heterogeneity (Fig. 3).

#### Prevalence of serious complications

**Fig. 4:**
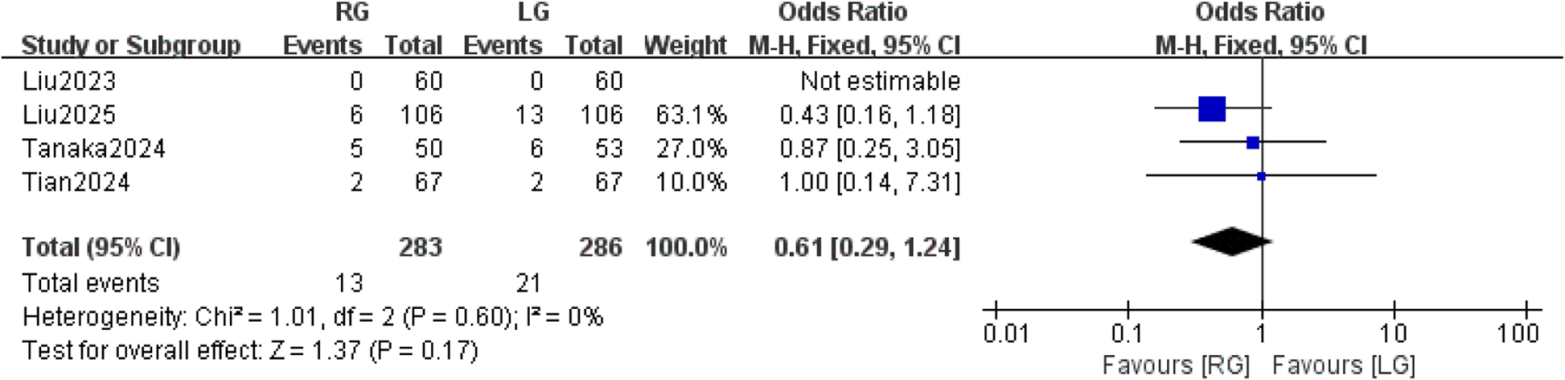
Prevalence of serious complications

Four studies [27–30] reported the prevalence of serious complications, which included grade 3 and 4 complications as defined by the Clavien-Dindo classification [31, 32]. The results of the heterogeneity test indicated no statistically significant heterogeneity among the studies (*P* = 0.60, I^2^ = 0%), using the fixed effects model. The meta-analysis revealed that the incidence of serious complications following RG and LG was similar, with no statistically significant difference observed [OR = 0.61, 95% CI: (0.29, 1.24), *P* = 0.17] (Fig. 4).

### Postoperative hospital stays

**Fig. 5:**
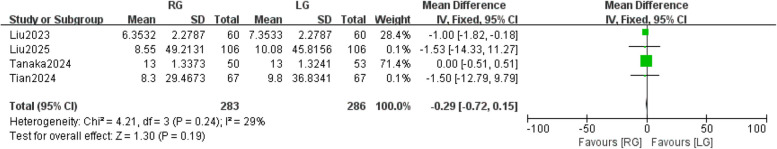
Postoperative hospital stays

A total of 569 patient participants in four studies [27–30] mentioned postoperative hospital stays. Fixed-effects meta-analysis showed no statistically significant difference in postoperative hospital stays between the RG and LG groups [MD: −0.29;95% CI: (−0.72,0.15), *P* = 0.19]. There was no significant heterogeneity between studies, which was (*P* = 0.24, I^2^ = 29%) (Fig. 5).

### Operative time

**Fig. 6:**
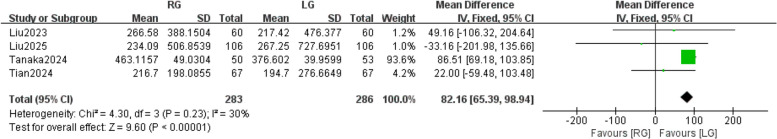
Operative time

Operative time was mentioned in all the total studies [27–30]. Fixed-effects meta-analysis showed a statistically significant prolongation of the operative time in the RG group compared to the LG group (MD 82.16;95%CI: 65.39,98.94, *P* < 0.00001). There was no significant heterogeneity between studies with (*P* = 0.23, I^2^ = 30%) (Fig. 6).

### Blood loss

**Fig. 7:**
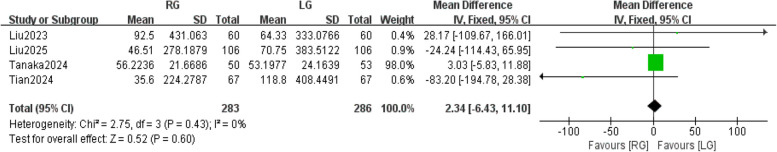
blood loss

Five hundred and sixty-nine patient participants in the four studies [27–30] mentioned intraoperative blood loss. Fixed-effects meta-analysis showed that the similarity in intraoperative blood loss between the RG and LG groups was not statistically significant (MD: 2.34; 95% CI: −6.43,11.10, *P* = 0.60). There was no significant heterogeneity between studies, which: (*P* = 0.43, I2 = 0%) (Fig. 7).

### Open conversion

**Fig. 8:**
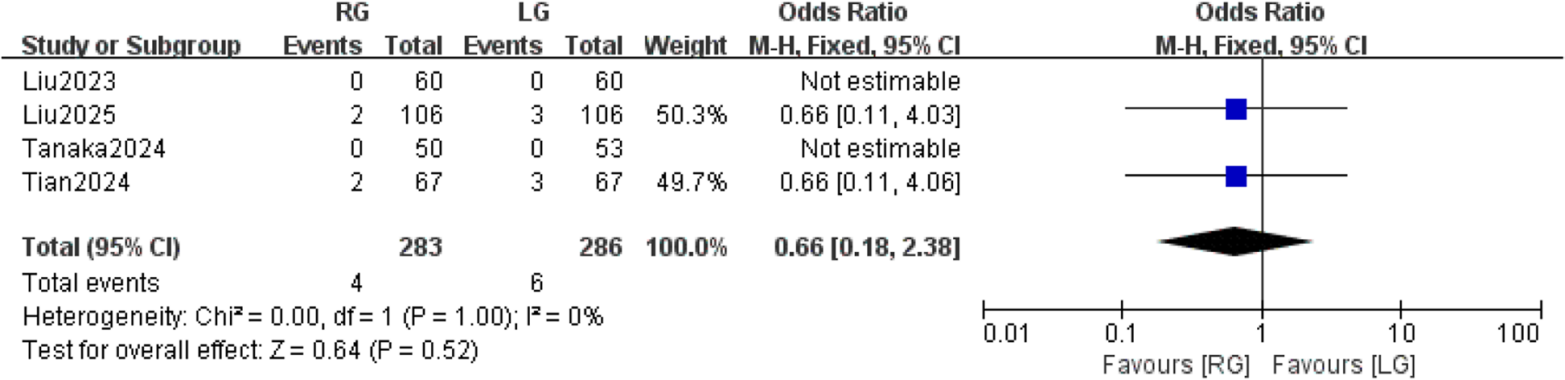
open conversion

Two studies [27, 28] reported the open conversion. Fixed-effects meta-analysis showed that the similarity in open conversion between the RG and LG groups was not statistically significant (OR: 0.66; 95% CI: 0.18,2.38, *P* = 0.52). There was no significant heterogeneity between studies with (*P* = 1.0, I^2^ = 0%) (Fig. 8).

### Number of lymph nodes

**Fig. 9:**
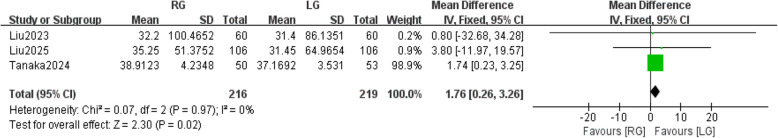
number of lymph nodes

A total of 435 patients in the three trials [28–30] reported the number of lymph nodes.Fixed-effects meta-analysis showed that the RG can harvest more the number of lymph nodes compared to the LG (MD: 1.76; 95% CI: 0.26,3.26, *P* = 0.02). There was no significant heterogeneity between studies (*P* = 0.97, I^2^ = 0%) (Fig. 9).

### The first flatus

**Fig. 10:**
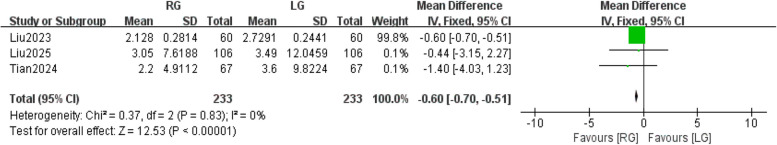
The first flatus

A total of 466 patient participants in the three studies [27, 28, 30] mentioned the first flatus. Fixed-effects meta-analysis showed a shorter time to first flatus in RG than LG (MD: −0.60;95% CI: −0.70, −0.51, *P* < 0.00001). There was no significant heterogeneity between the studies with (*P* = 0.83, I^2^ = 0%) (Fig. 10).

### The first time on liquid diets

**Fig. 11:**
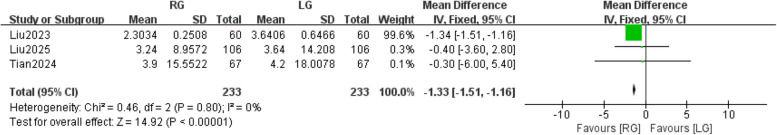
The first time on liquid diets

A total of 466 patient participants in the three studies [27, 28, 30] indicated the first time on liquid diets. The fixed effects meta-analysis showed a statistically significant shorter time to first time on liquid diets in RG than LG. (MD: −1.33; 95% CI: −1.51, −1.16, *p* < 0.00001). There was no significant heterogeneity between studies (*P* = 0.80, I^2^ = 0%) (Fig. 11).

### Reoperation within 30 days after surgery

**Fig. 12:**
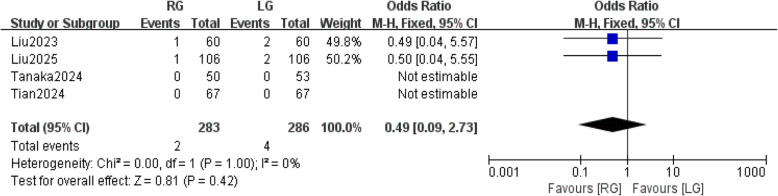
reoperation within 30 days after surgery

Two studies [28, 30] reported on reoperation within 30 days after surgery. Fixed-effects meta-analysis showed that there was no statistically significant difference in reoperation within 30 days after surgery between the RG and LG groups (OR: 0.49;95% CI:0.09,2.73, *P* = 0.42). There was no significant heterogeneity between studies (*P* = 1, I2 = 0%) (Fig. 12).

### Readmission within 30 days after surgery

**Fig. 13:**
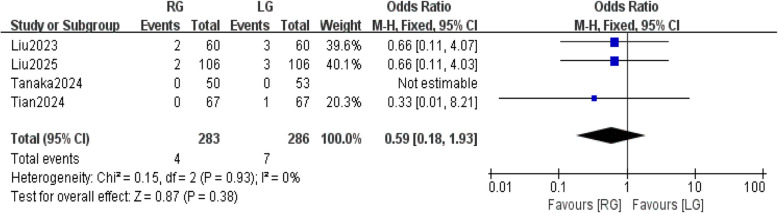
Readmission within 30 days after surgery

Three studies s [27, 28, 30] involved readmission within 30 days after surgery. Fixed-effects meta-analysis showed that between the RG and LG groups, there was no statistically significant difference in readmission within 30 days after surgery (OR: 0.59; 95% CI: 0.18,1.93, *P* = 0.38). There was no significant heterogeneity between studies (*P* = 0.93, I2 = 0%) (Fig. 13).

## Discussion

The impact of robotic gastrectomy (RG) surgery on advanced gastric cancer following neoadjuvant therapy remains a topic of debate. We included four non-randomized studies from East Asia, all involving advanced (II-III) gastric cancers that underwent RG following neoadjuvant chemotherapy. Our findings indicate that RG had a longer operating time than LG, but more lymph nodes were harvested. Furthermore, the first time on liquid diets, the first flatus, was significantly shorter compared to LG. Simultaneously, there was no statistically significant difference in the postoperative complications and serious postoperative complications, postoperative hospital stays, blood loss, readmission within 30 days after surgery, secondary surgery within 30 days after surgery, open conversion. Postoperative complications represent a critical component of evaluating short-term outcomes following RG. Complications can seriously affect postoperative recovery and the practical performance of surgery. Our study's findings demonstrated that RG and LG exhibited comparable outcomes with no substantial difference in postoperative complication rates. However, a degree of heterogeneity was observed among the studies. To identify the source of this heterogeneity, we conducted a further sub analysis of institutional surgical experience. The findings of the study indicated that there was statistically significant difference between the two groups. The observed heterogeneity in postoperative complications may be attributed to the institutional surgical experience, with the presence of heterogeneity being deemed acceptable. As the study was retrospective and had some reporting and selection bias, which affected the outcome to some extent, this needs verification in future multicenter randomized trials. In our study, we found that surgeons spent more time on RG compared to LG. But we admitted this is a known objective result [33]. Laparoscopic surgery is now routinely performed by the general surgeon is experienced, while robotic surgery as an emerging technology surgeons still lack a certain experience is also the main reason for limiting the extension of operation time, there is a certain amount of experience in laparoscopic surgery surgeons will be able to master the robotic surgery, with the completion of the learning curve of the surgeon in charge of the learning curve and the ability to improve team cooperation, the time spent can be reduced [34, 35]. In terms of intraoperative blood loss, the results of our meta-analysis showed no significant difference between robotic and laparoscopic similarity. Open conversion in minimally invasive surgery is important for two reasons. First, it shows how complex the procedure is. Second, it has important clinical value and research implications. A meta-analysis that included 25,521 patients showed that there was no significant difference between RG and LG in terms of open conversion [36]. Our analysis of the data confirmed these findings. The first flatus and the first time on liquid diets are important indicators that show how well the gastrointestinal system is recovering and are also important ways to assess how quickly patients are recovering in the early postoperative period [37, 38]. Our meta-analysis showed that the first flatus and the first time on liquid diets were a lot shorter compared with LG. There are a few possible reasons for this. First, the robotic system provides a stable operating platform. This helps to avoid unnecessary stimulation and straining during surgery. On the other hand, in recent years, the idea of Enhanced Recovery After Surgery (ERAS) has been used more and more in gastrointestinal surgery. This idea supported that patient could move early after surgery [39, 40], which helps them recover their gastrointestinal function. In the future, higher-level RCT studies will be needed to confirm the effect of robotics on the recovery of gastrointestinal function. However, a review of the research showed that the factors that could have affected the results did not lead to different lengths of time for patients in the RG and LG groups to stay in the hospital after surgery. There was no statistical difference in how long people stayed in the hospital after surgery between the two groups, and the results seemed to favor the RG. Oncologic outcomes, the lymph node dissection steps for RG are essentially the same as those for LG, and the number of lymph nodes harvest not only determines the accuracy of postoperative staging assessment but also relates to the patient's prognosis [41, 42]and is one of the independent risk factors for recurrence and metastasis of gastric cancer [43, 44]. Also, the results of our meta-analysis showed that in agreement with the above evidence.A recent study of risk factor analysis showed a significant association between readmission after discharge and postoperative complications in patients after gastrectomy [45]. Our current meta-analysis results show that there is no significant difference between RG and LG readmission within 30 days after discharge from the hospital. In general, reoperation within 30 days after surgery is aimed at saving lives and halting the progression of disease. Anastomotic leakage, stump leakage, severe infection, intestinal obstruction, and active bleeding are the primary reasons for reoperation within 30 days after surgery [46]. Our meta-analysis shows that reoperation within 30 days after surgery, RG and LG are similar and not significantly different. Our study possesses the following strengths: First, we performed an extensive database search without restrictions on language or time. Second, we incorporated the most recent research data, and the experiments included were conducted in the top hospitals, thereby enhancing the reliability of the pooled results. The current meta-analysis is subject to several methodological limitations: Since the four included studies were retrospective, they were inherently subject to selection bias and reporting bias, undermining the statistical robustness of pooled effect estimates. All studies originated from East Asian populations (Japan and China), raising concerns about external validity. Regional variations in genetic profiles, clinical practices, and protocols may limit the generalizability of findings to non-Asian populations. What is more, we could not analyze the data together for long-term outcomes and costs due to inconsistencies in the standards used across studies. Future research should focus on high-quality prospective studies and RCTs to further explore the RG`s long-term outcomes and costs in advanced gastric cancer patients undergoing neoadjuvant therapy. Definitive conclusions require validation through multicenter randomized controlled trials with standardized surgical protocols. Clinicians should exercise caution when extrapolating these results to broader populations until higher-level evidence is available.

## Conclusion

In conclusion, the result suggests that robotic surgery after neoadjuvant therapy is a treatment approach with great potential for development and may be used as a new treatment method for locally advanced gastric cancer. More randomized clinical trials remain essential further to demonstrate the value of robotic surgery for advanced gastric cancer after neoadjuvant therapy.

## Supplementary Information

Supplementary Material 1.

Supplementary Material 2.

Supplementary Material 3.

## Data Availability

All data generated or analysed during this study are included in this published article.

## Abbreviations

RG: Robotic gastrectomy
LG: Laparoscopic gastrectomy
CNKI: Chinese National Knowledge Infrastructure
MD: Mean difference
CI: Confidence interval
OR: Odds ratio
RCT: Randomized controlled trials
pCR: Complete pathologic response
ERAS: Enhanced Recovery After Surgery
PSM: Propensity score matching
BMI: Body mass index
Mesh: Medical subject headings
SOX: S-1 plus Oxaliplatin
XELOX: Xeloda Plus Oxaplatin
DOS: Docetaxel Plus Oxaplatin Plus S-1
FLOT: Fluorouracil (5-FU) Plus Leucovorin Plus Oxaliplatin Plus Docetaxel
