# Supplementary material for: Short-term outcomes of robotic vs. laparoscopic surgery for gastric cancer after neoadjuvant therapy: a systematic review and meta-analysis
Source: BMC Cancer. 2025 Jun 5;25:1002. doi: 10.1186/s12885-025-14395-3 (PMC12139109; doi:10.1186/s12885-025-14395-3)
Supplement: Supplementary file 1 — Supplementary Material 1. [file 12885_2025_14395_MOESM1_ESM.docx]

Figure1

**Identification of studies via databases and registers**

Records removed *before screening*:

Duplicate records removed

(n =23)

Records identified from*:

Databases (n = 139)

**Identification**

Records screened

(n = 116)

Articles excluded after review of the title and abstracts (n =53)

Reports sought for retrieval

(n = 63)

Reports not retrieved

(n = 0)

**Screening**

Reports excluded:
1. Reviews, conference abstracts letters to the editor, case reports and technical reports (n = 58)

2.The absence of key data (n =1)

Reports assessed for eligibility

(n = 63)

Studies included in review

(n = 4)

**Included**

Flow chart of literature search and screening.
