# Supplementary material for: Short-term outcomes of robotic vs. laparoscopic surgery for gastric cancer after neoadjuvant therapy: a systematic review and meta-analysis
Source: BMC Cancer. 2025 Jun 5;25:1002. doi: 10.1186/s12885-025-14395-3 (PMC12139109; doi:10.1186/s12885-025-14395-3)
Supplement: Supplementary file 2 — Supplementary Material 2. [file 12885_2025_14395_MOESM2_ESM.docx]

**Supplementary table 1.** Detailed search strategies of all target databases.

*Search strategy of Pubmed*

| No. | Search term | Results |
| --- | --- | --- |
| #1 | stomach neoplasm OR gastric neoplasm OR stomach cancer OR gastric cancer OR stomach tumor | #187712 |
| #2 | laparoscopic OR laparoscopy OR laparoscope OR Laparoscopic surgery | #188999 |
| #3 | robotic OR robotics OR robotic-assisted OR robot-assisted OR Davinci robot) | #112451 |
| #4 | neoadjuvant OR neoadjuvant chemoradiotherapy OR neoadjuvant chemotherapy OR neoadjuvant radiotherapy | #65048 |
| #5 | #1 AND #2 AND #3 AND #4 | #49 |
|  |  |  |

| *Search strategy of Embase* | | |
| --- | --- | --- |
| No. | Search details | Results |
| #1 | stomach tumor'/exp | # 219782 |
| #2 | stomach tumor’: ab, ti OR ('gastric mass':ab,ti AND tumor: ab,ti) OR 'gastric neoplasia':ab,ti OR 'gastric tumor':ab,ti OR 'tumour of the stomach':ab,ti OR 'gastric cancer':ab,ti OR 'stomach neoplasm':ab,ti OR 'gastric neoplasm':ab,ti | #121143 |
| #3 | #1 OR #2 | #231256 |
| #4 | laparoscopic surgery'/exp | #134711 |
| #5 | 'laparoscopic surgery’: ab, ti OR laparoscopic: ab, ti OR laparoscopy:ab,ti OR laparoscope: ab,ti | #266414 |
| #6 | #4 OR #5 | #294949 |
| #7 | neoadjuvant therapy'/exp | #68455 |
| #8 | neoadjuvant therapy’: ab,ti OR 'neoadjuvant treatment':ab,ti OR 'neoadjuvant chemotherapy':ab,ti OR 'neoadjuvant chemoradiotherapy':ab,ti OR 'neoadjuvant systemic treatments':ab,ti OR 'neoadjuvant radiotherapy':ab,ti | #75806 |
| #9 | #7 OR #8 | #102924 |
| #10 | robot assisted surgery'/exp | #41194 |
| #11 | robot surgery':ab,ti OR 'robot assisted surgery':ab,ti OR 'robotic surgical procedure':ab,ti OR robotic: ab,ti OR robotics: ab,ti | #93255 |
| #12 | #10 OR #11 | #107148 |
| #13 | #3 AND #6 AND#9 AND #12 | #78 |

| *Search strategy of Cochrane library* | | |
| --- | --- | --- |
| No. | Search Details | Results |
| #1 | Mesh descriptor: [Neoadjuvant Therapy] explode all trees | 2729 |
| #2 | (NeoadjuvantTherapy)OR(Neoadjuvant Treatment) OR(neoadjuvant chemoradiotherapy) OR(neoadjuvant chemotherapy)OR(Neoadjuvant Systemic Treatments) | 13637 |
| #3 | #1OR#2 | 13637 |
| #4 | MeSH descriptor: [Stomach Neoplasms] explode all trees | 4153 |
| #5 | (Stomach Neoplasm)OR(Gastric Neoplasm)OR(Cancer of Stomach)OR(Stomach Cancer)OR(Gastric Cancer)OR(Stomach tumor) | 12567 |
| #6 | #4OR#5 | 12806 |
| #7 | MeSH descriptor: [Robotics] explode all trees | 2077 |
| #8 | (robotics)OR（robotic-assisted）OR（Robotic-Assisted Surgeries）OR（Davinci robot）OR（Robotic Surgical） | 4843 |
| #9 | #7 OR #8 | 4861 |
| #10 | MeSH descriptor: [Laparoscopy] explode all trees | 9244 |
| #11 | (Laparoscopic)OR(Laparoscopy)OR(laparoscope)OR (Laparoscopic Assisted Surgeries)OR(Laparoscopic Surgical Procedures)OR(Laparoscopic Surgery) | 30168 |
| #12 | #10 OR#11 | 30558 |
| #13 | MeSH descriptor: [Randomized Controlled Trial] explode all trees | 34 |
| #14 | MeSH descriptor: [Randomized Controlled Trials as Topic] explode all trees | 60261 |
| #15 | MeSH descriptor: [Random Allocation] explode all trees | 25497 |
| #16 | #13 or #914or #15 | 85542 |
| #17 | #3 and #6 and #9and #12and#16 | 0 |

| *Search strategy of Web of Science* | | |
| --- | --- | --- |
| No. | Search Query | Results |
| #1 | Gastric Cancer (Topic) OR Stomach Neoplasm (Topic) OR Gastric Neoplasm (Topic)OR Stomach Cancer (Topic)OR Stomach tumor (Topic) | 117295 |
| #2 | Laparoscopic Assisted Surgeries (Topic) OR Laparoscopic Surgical Procedures (Topic) OR Laparoscopic (Topic) OR Laparoscopy (Topic) OR laparoscope (Topic) OR Laparoscopic(Topic) | 125347 |
| #3 | Combined Modality Therapy(Topic) Neoadjuvant Therapy(Topic) Neoadjuvant Treatment (Topic) neoadjuvant chemoradiotherapy (Topic) neoadjuvant chemotherapy (Topic) neoadjuvant radiotherapy (Topic) Neoadjuvant Systemic Treatments(Topic) | 77794 |
| #4 | Robotic (Topic) OR robotics (Topic) OR robotic-assisted (Topic) OR Robotic-Assisted Surgeries (Topic) OR Robotic Surgical (Topic) OR Davinci robot system (Topic) | 118988 |
| #5 | random* (All Fields) | 63662 |
| #6 | #1AND #2 AND #3 AND #4 AND #5 | 0 |

| *Search strategy of Chinese National Knowledge Infrastructure (CNKI)* | | |
| --- | --- | --- |
| No. | Search Details | results |
| #1 | (Topic: Laparoscopic assisted [precise]) OR (Topic: Laparoscopic [precise]) OR (Topic: Laparoscopy [precise]) |  |
| #2 | (Topic: gastric cancer [precise]) |  |
| #3 | (Topic: Robotic [precise]) OR (Topic: robotic-assisted [precise]) OR (Topic: Davinci robot [precise]) |  |
| #4 | (Topic: Neoadjuvant Therapy [precise]) OR (Topic: neoadjuvant chemotherapy [precise]) OR (Topic: neoadjuvant radiotherapy [precise]) |  |
| #5 | (All Fields: random control trails [precise]) |  |
| #6 | #1 and #2 and #3and#4and#5 | 12 |
